# Potential Use of Porous Titanium–Niobium Alloy in Orthopedic Implants: Preparation and Experimental Study of Its Biocompatibility In Vitro

**DOI:** 10.1371/journal.pone.0079289

**Published:** 2013-11-19

**Authors:** Jian Xu, Xiao-Jun Weng, Xu Wang, Jia-Zhang Huang, Chao Zhang, Hassan Muhammad, Xin Ma, Qian-De Liao

**Affiliations:** 1 Department of Orthopedics, Huashan Hospital, Fudan University, Shanghai, China; 2 Department of Joint Surgery, Hunan Provincial People’s Hospital, Hunan Province, China; 3 Department of Orthopedics, Xiangya Hospital, Central South University, Hunan Province, China; University of Akron, United States of America

## Abstract

**Background:**

The improvement of bone ingrowth into prosthesis and enhancement of the combination of the range between the bone and prosthesis are important for long-term stability of artificial joints. They are the focus of research on uncemented artificial joints. Porous materials can be of potential use to solve these problems.

**Objectives/Purposes:**

This research aims to observe the characteristics of the new porous Ti-25Nb alloy and its biocompatibility in vitro, and to provide basic experimental evidence for the development of new porous prostheses or bone implants for bone tissue regeneration.

**Methods:**

The Ti-25Nb alloys with different porosities were fabricated using powder metallurgy. The alloys were then evaluated based on several characteristics, such as mechanical properties, purity, pore size, and porosity. To evaluate biocompatibility, the specimens were subjected to methylthiazol tetrazolium (MTT) colorimetric assay, cell adhesion and proliferation assay using acridine staining, scanning electron microscopy, and detection of inflammation factor interleukin-6 (IL-6).

**Results:**

The porous Ti-25Nb alloy with interconnected pores had a pore size of 200 µm to 500 µm, which was favorable for bone ingrowth. The compressive strength of the alloy was similar to that of cortical bone, while with the elastic modulus closer to cancellous bone. MTT assay showed that the alloy had no adverse reaction to rabbit bone marrow mesenchymal stem cells, with a toxicity level of 0 to 1. Cell adhesion and proliferation experiments showed excellent cell growth on the surface and inside the pores of the alloy. According to the IL-6 levels, the alloy did not cause any obvious inflammatory response.

**Conclusion:**

All porous Ti-25Nb alloys showed good biocompatibility regardless of the percentage of porosity. The basic requirement of clinical orthopedic implants was satisfied, which made the alloy a good prospect for biomedical application. The alloy with 70% porosity had the optimum mechanical properties, as well as suitable pore size and porosity, which allowed more bone ingrowth.

## Introduction

Aseptic loosening can cause failure of artificial joint replacement. However, loosening can be prevented by improving the stability of the bone–implant interface, which depends on the combined strength between the bone and biomaterials. Osseointegration is closely related to the mechanical features and surface characteristics of implants. The surfaces of artificial joint prostheses are always treated with hydroxyapatite coating and rough surfaces, resulting in good integration of the bone and implant. However, this kind of treatment limits the combination of prosthesis and bone strength, as well as depth and scope, with a bone union rate of only 6% to 20% [1], [2], [3]. Therefore, improving the bone ingrowth into prosthesis and enhancing the combination of the range between bone and prosthetic surface are important for long-term stability of artificial joints and the focus of research on uncemented artificial joints.

Titanium has excellent physical properties and biocompatibility [4]. The titanium–niobium alloy is nontoxic and has good machinability and mechanical strength. The alloy has an elastic modulus that is close to the bones and superior corrosion resistance than pure titanium. Therefore, the alloy can be used as a biomedical material for orthopedic applications. Though Ti with a porosity of 70% which exhibited a plateau stress of 53 MPa and an elastic modulus of 3.4 GPa [5]. Nevertheless, its compressive strength is still lower compared with cortical bone [6]. A porous Ti-6Al-4V alloy scaffold was reported to obtain higher strength than porous Ti at the same porosity [7], [8]. Porous Ni and TiNi also presented good mechanical properties [9], [10]. However, the release of metal ions from some metal materials, e.g. aluminium (Al), nickel (Ni), iron (Fe),vanadium (V) and chromidium (Co), can generate adverse biological effects [11]. Some research on the biological behaviour of metals had shown the composition of implant biomaterials must be carefully selected to avoid or minimize adverse reactions [11]. On the other hand, titanium (Ti), niobium (Nb) and tantalum (Ta) are believed to be non-toxic metals with good biocompatibility [12]. Compared with Ta, titanium (Ti) and niobium (Nb) are lightweight and cheaper material and yet had displayed an exceptional biocompatibility in orthopedic and dentistry literature [13]. There is little research on porous Ti-Nb binary alloys. We used powder metallurgy (PM) which was a useful technology for the production of small parts with a complicated shape [14] to fabricate porous titanium–niobium alloy samples with different porosities and a mechanical strength similar to cortical bone. Our aim was to observe the characteristics of Ti-25Nb alloy and assess its biocompatibility, thereby providing experimental foundation for the clinical application of porous Ti-25Nb alloys.

## Materials and Methods

All animal experiments were performed according to protocols approved by the Institutional Animal Care Committee of Fudan University. This research consisted of the preparation and biocompatibility evaluation of porous Ti-25Nb alloy.

### Preparation of Ti-25Nb

**Figure 1:**
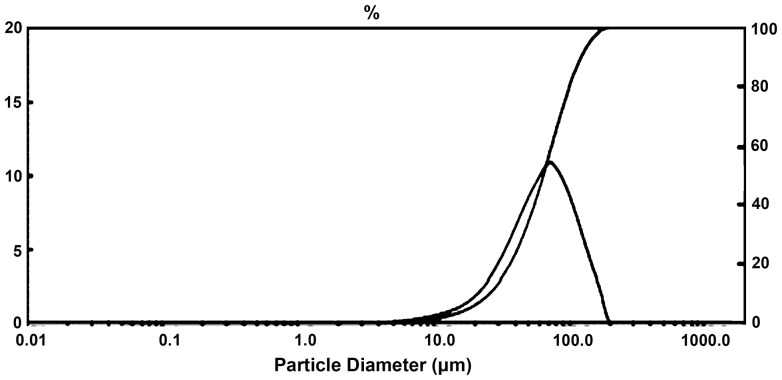
Particle size of titanium powder by analysis of laser.

**Figure 2:**
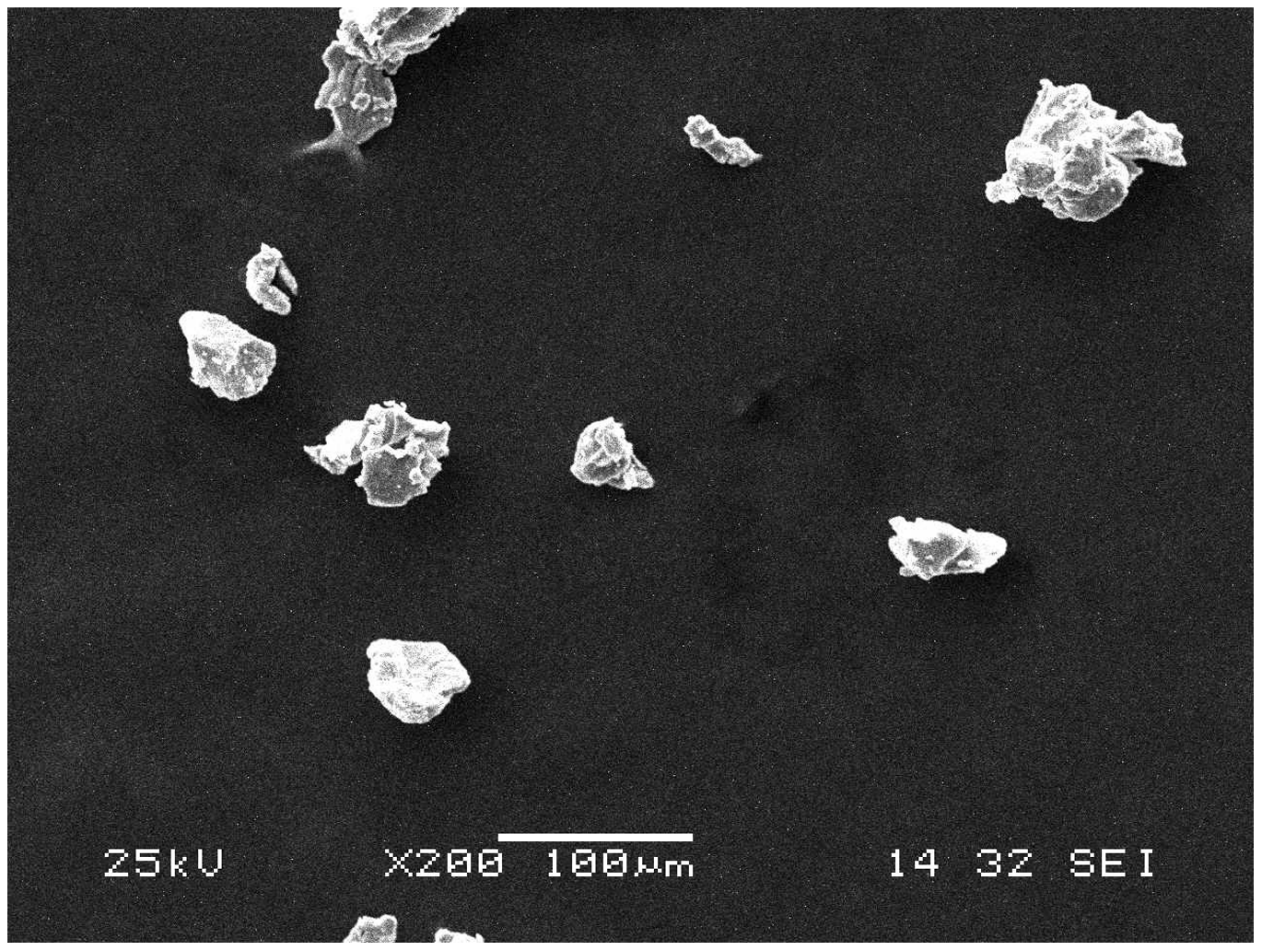
Scanning electron micrograph of titanium powder.

**Figure 3:**
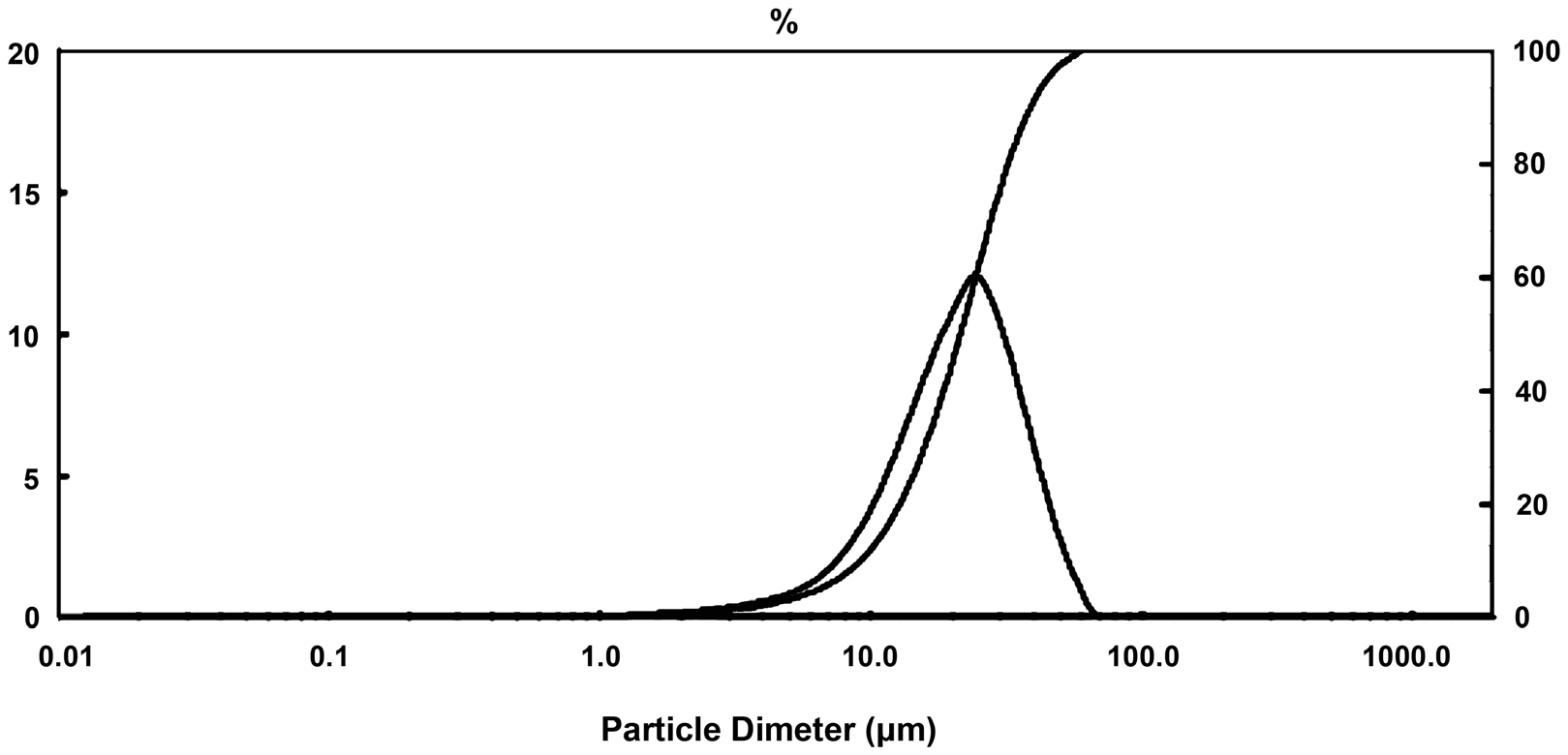
Particle size of niobium powder by analysis of laser.

**Figure 4:**
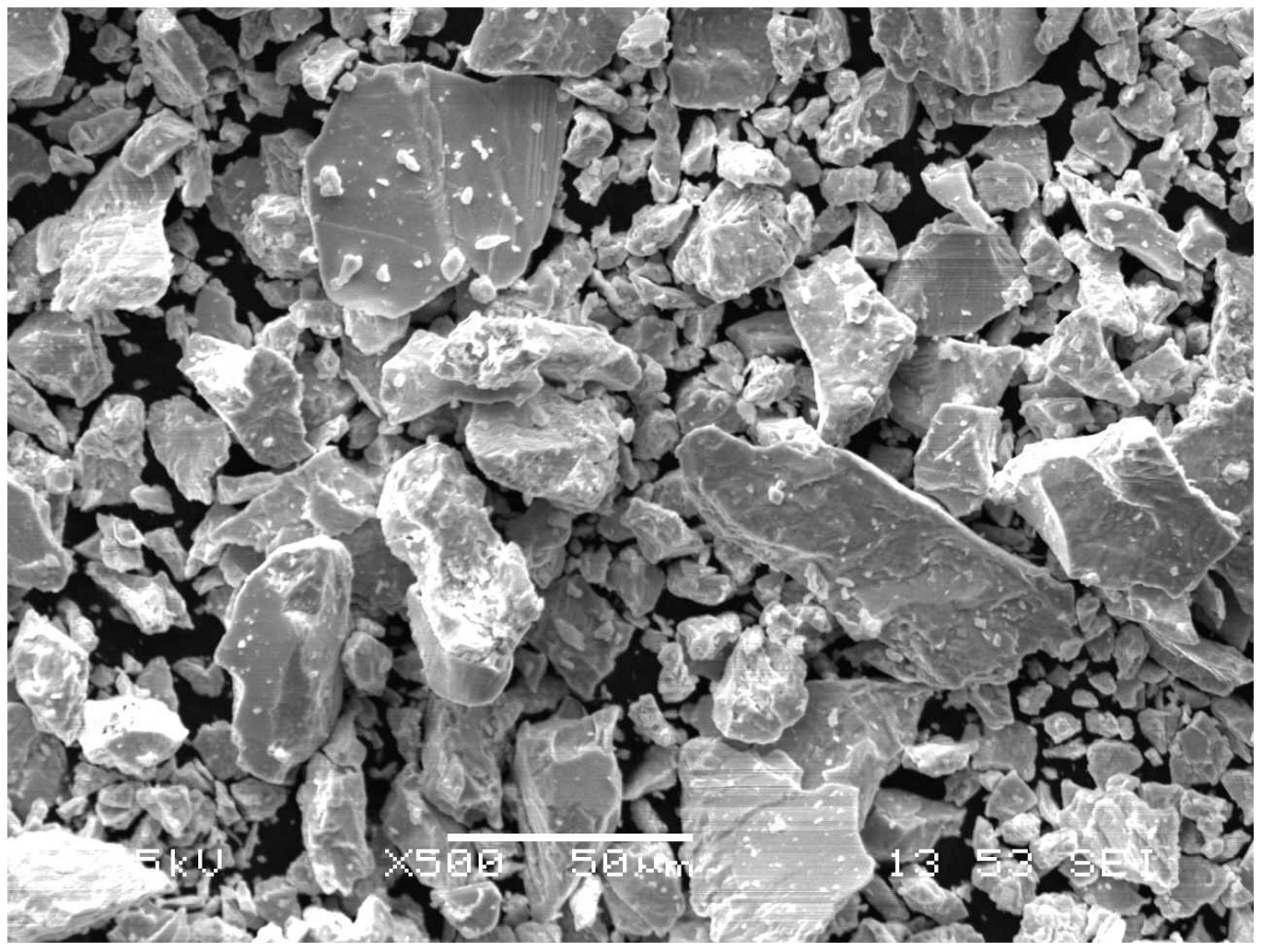
Scanning electron micrograph of niobium powder.

This research adopted PM. The titanium powder and niobium powder which was purchased from Zhuzhou Chemical Reagent Co. (China) was 99.7% and 99.5% respectively in purity (Table1 and Table2) by an average grain diameter of Ti (64 µm) and Nb (31.52 µm),with the range of 32.65 µm - 79.13 µm(Ti) (Fig. 1 and Fig. 2) and 7.72 µm-41.63 µm(Nb) (Fig. 3 and Fig. 4). Both powders were maldistributed. Elemental metal powders of Ti and Nb were weighed to give a nominal composition of Ti-25Nb (hereafter wt.%).

**Table 1:** Original performance parameters of titanium powder.

Powder	Melting point (°C)	Purity (%)	Average grain diameter (µm)
Titanium powder	1660	≥99.7	64 µm

**Table 2:** Main composition of niobium powder (x%).

Elements	Nb	Ta	O	N	C	Fe
Content	99.4965	0.0500	0.3600	0.0400	0.0440	0.0065

**Table 3:** Parameters of the polyurethane foam.

Foam type	Density	Pore size
DYMJP/R2D25/XYa	25 g/cm^3^	1 mm to 2 mm

**Table 4:** Performance parameters of polyurethane.

Type	Density (Kg/m^3^)	Pore size (mm)
Polyurethane (50 PPI to 70 PPI)	50–70	0.593–0.847

The organic foam scaffolds in the experiment was celled polyurethane foam in R2D25 type (Changzhou, China). The detailed parameters were listed in Table3 and Table4. The agglomerant was polyving akohol (PVA) (Tianjing, China) which had a molecular formula as [C_2_H_4_O]n, with an average relative molecular weight 127745 and 97% in purity. Its performance parameters were listed in Table 5.

**Table 5:** Performance parameters of polyving akohol (PVA).

Name	Molecular formula	Relative molecular weight	Purity
PVA	[C_2_H_4_O]_n_	127745	97.0%

**Figure 5:**
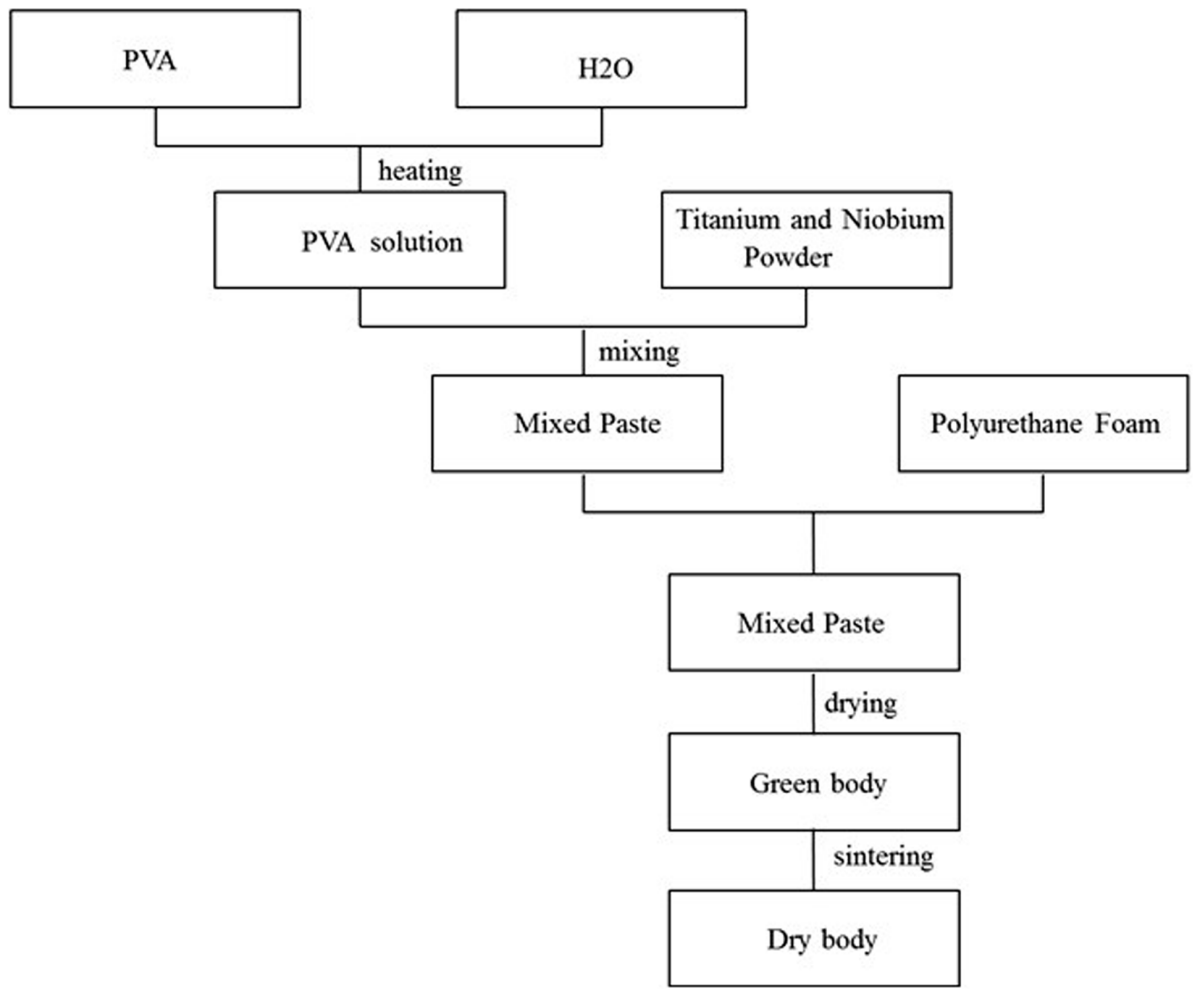
Flow chart of preparation of porous titanium-niobium alloy.

The powder was prepared according to the experimental design, and mixed for 4 h in a planetary grinding machine to obtain a completely uniform mixture. Firstly a certain amount of polyvinyl alcohol was put into the distilled water and heated to be dissolved. After it cooled down, take a certain amount of solution added to titanium niobium powder and mixed them into paste fully. Every 100 g of the titanium niobium powder were mixed with suitable amount of polyvinyl alcohol solution. Make use of the foam to dip into the solution, as long as it was completely dipped then moved it into the vacuum drying oven for drying, in which the temperature should be ranged from 70 to 80 centigrade, the lasted time 2 to 4 hours and the vacuum degree below 5 Pa. The sintering temperature for the first time ranged from 1400°C to 1500°C with argon gas as the protective atmosphere, keeping the lasting time for 2 to 3 hours, and then continue to heat up for the second sintering with a higher temperature from 1700–1800°C and the lasting time kept for 1–2 hours. After that, take out of the specimens for postprocessing including clearing the surface contaminants. The preparation process is shown in Fig. 5.

### Characterization of Porous Ti-25Nb Alloy

A scanning electron microscopy (SEM) (JSM5600-LV, Japan) equipped with an X-ray energy-dispersive spectrometer (EDS) (Oxford Instruments, UK) was employed to qualitatively determine the morphological characteristics, pore size and composition analysis of Ti-25Nb alloy. Appropriate scanning photographs were chosen. Each sample was observed for three selected visions. Ten aperture values were measured in each vision. Then, the average pore diameter values were calculated.

To determine the porosity, mercury injection method was used for porosity determination. The porosity of titanium–niobium alloy was calculated using the formula: ε = 1−ρ/ρ_0_, where ρ is the density of porous titanium–niobium alloy and ρ_0_ is the theoretical density of titanium–niobium alloy (8.57 g/cm^3^). However, the opening porosity is commonly adopted in practical applications and is measured by the liquid penetrating method [15], [16]. Porosity factor calculations were made using the following equations [17], [18]: V_d_ = (m_d_−m_d_′)/ρ_w_ V_s_ = (m_s_−m_s_′)/ρ_w_ PF = (V_s_−V_d_)×100/V_d._

Where: V_d_ = dried specimen volume; m_d_ = mass of dried specimen in air; m_d_′ = mass of dried specimen in water; ρ_w_ = density of water; V_s_ = volume of the specimen saturated with water; m_s_ = mass of saturated specimen in air and m_s_′ = mass of saturated specimen in water. PF = porosity. In the first and second equations, the volumes were determined, using the following known values obtained by weights and the ρ_w_ = 1000 Kg/m^3^.

### Measurement of Mechanical Properties on Porous Ti-25Nb Alloy

Compressive strength was obtained by unidirectional compression using cylindrical samples (the length-to-diameter ratio was 2.5∶1 to 3.5∶1). Strength was obtained using the following formula: σ = F_max_/A_0_, where F_max_ is the destruction force for the sample, the compressive strength σ is the maximum stress in the process of sample destruction, and A_0_ is the cross-sectional area of the impact site. After compression tests in a electronic universal testing machine (CMT4000, Japan), the compression strength and elastic modulus of samples with different porosities were compared and analyzed.

### Biocompatibility of Porous Titanium–niobium Alloy

#### Cell seeding and co-culture of samples

All samples were sterilized in ethylene oxide gas before cell seeding. Rabbit bone marrow mesenchymal stem cells (BMMSCs) were isolated from newborn New Zealand rabbits [19]. BMMSCs were identified by flow cytometry (FACS) (Becton-Dickinson, USA). Cells were cultured in Dulbecco’s modified eagle medium (DMEM) (Gibco, USA) supplemented with 10% fetal bovine serum (FBS) and 1% penicillin/streptomycin, and incubated at 37°C in a 5% CO_2_ and 100% relative humidity incubator. The third passage cells were used in this study. BMMSCs were seeded on the samples at a density of 6×10^5^ cells/mL.

#### Cell adhesion and proliferation test

**Figure 6:**
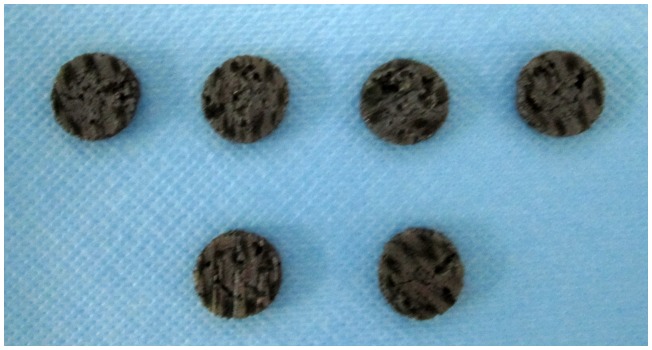
The titanium-niobium alloy specimens with different porosity.

The samples were processed into disc-shaped specimens (1 mm thick and 10 mm diameter) (Fig. 6). The samples were divided into three groups, namely, compact group, 40% porosity group, and 70% porosity group. All samples were prepared for the tests using the following rinsing process: ultrasonic cleaning in distilled water, acetone solution, 70% ethanol solution, and distilled water for 20, 20, 20, and 15 min,app:addword:acetone solution respectively. Finally, the samples were treated with 75% ethanol solution. Samples were disinfected at high temperature and high pressure for 30 min before analysis.

#### SEM observation

After the cells were cleaned, fixed, and dried, cell morphology was observed using SEM.

#### Cytotoxicity assay

Cytotoxicity was assessed using methylthiazol tetrazolium (MTT) (Gibco, USA) assay. 15 samples were prepared, with 5 samples in each of the three groups. The leaching solution was prepared by mixing the samples of different groups with the cell culture medium (10% FBS+DMEM/F12+ BFGF) (Gibco, USA). The solution was then diluted to obtain 100% and 50% concentrations. The suspension of third-generation BMSCs was prepared after trypsin digestion, and the cell concentration was adjusted to 1×10^5^/mL. A total of 200 µL of leaching solution with different concentrations (50% and 100%) was added to each well for the experimental groups. For the blank control group, 200 µL of cell culture medium was added to each well.

MTT assay was conducted by culturing the samples for 2, 4, and 7 d. Four hours before culture termination, 20 µL of 0.5% MTT was added to each well, and the culture was incubated for 4 h. The culture medium was then sucked out, and 150 µL dimethyl sulfoxide (Gibco, USA) was added to the wells. The cultures were kept at room temperature for 15 min to 20 min. The culture plate was shaken for 10 min for uniform dyeing. An enzyme-linked detector was used at 492 nm wavelength for each well to calculate the average OD of each group, with the reference wavelength of 655 nm.

In the evaluation, the average OD values of 100% and 50% concentrations of leaching solution were calculated with the OD value of the blank control group as the 100% cell proliferation rate. The cell proliferation percentage (P%) of each concentration group was calculated according to the formula: P% = the mean OD of each concentration group/the average OD in blank control group × 100%. According to Table 6, P% of leaching solution of different concentrations was transformed into cell toxicity levels of 0 to 5.

**Table 6:** Corresponding relationships between cell growth rate and grade of cell toxicity.

Cell growing rate	Grade of cell toxicity
≥100%	0
75–99	1
50–74	2
25–49	3
0–24	4
0	5

#### Detection of Interleukin 6 (IL-6)

25 samples were prepared, with 5 samples in each of the five groups. For IL-6 detection, the specimens of Ti-6Al-4V, Ti-6Al-7Nb, dense type Ti-25Nb and Ti-25Nb with 40% and 70% porosity were included for comparison. In rabbit BMMSCs composited with the alloy samples, the supernatant liquid of the culture medium in the mixture were collected at each day of 0 to 7 and the time point of 2 w to detect the IL-6 content. Prior to analysis, the supernatant of the cell culture medium was stored in a refrigerator at −20°C. The kit contents and the supernatant of the cell culture medium were then preheated to room temperature. The reaction reagent was prepared according to the manufacturer’s instruction, including washing with buffer, standard dilution buffer, standard and quality control liquid, IL-6, IL-6 antibodies, and horseradish peroxidase fluid. The experiment was conducted according to the kit’s instructions (Cusabio, USA). The IL-6 concentration of each well was recorded and analyzed.

### Statistical Analysis

All statistical analyses were performed by SPSS20.0 software package (IBM, USA). Quantitative data are expressed as mean ± standard deviation. Variance analysis was used to compare the differences between groups at the same time point. Values of P<0.05 were considered significant, whereas values of P<0.01 were considered highly significant.

## Results

### Pore Size and Porosity of Titanium–niobium Alloy

**Figure 7:**
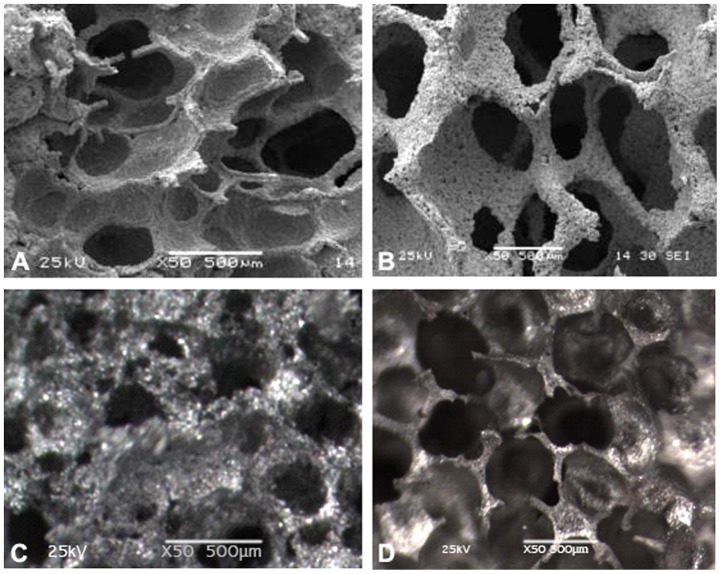
Pore size and porosity of Ti-25Nb alloy. (A)The titanium-niobium alloy formed porous shape with a rough surface, and its pores extended to the internal with a diameter of about 200–500 µm. (B) The pores interconnected with each other. (C) The pore of titanium-niobium alloy with 40% porosity showed maldistribution (D) The pore of titanium-niobium alloy with 70% porosity showed honeycomb shape, among which also presented part of fractures and connectivity.

The Ti-25Nb alloy material formed a porous structure with a rough surface after high-temperature sintering in a vacuum. This structure had a diameter of about 200 µm to 500 µm (Figs. 7A to 7D). A three-dimensional connectivity was observed between the pores. The pores of the Ti-25Nb alloy with 70% porosity showed a honeycomb structure with fractures and connectivity. The pores of the titanium niobium alloy with 40% porosity showed maldistribution with slight connectivity.

### EDS Analysis of Porous Ti-25Nb Alloy

**Figure 8:**
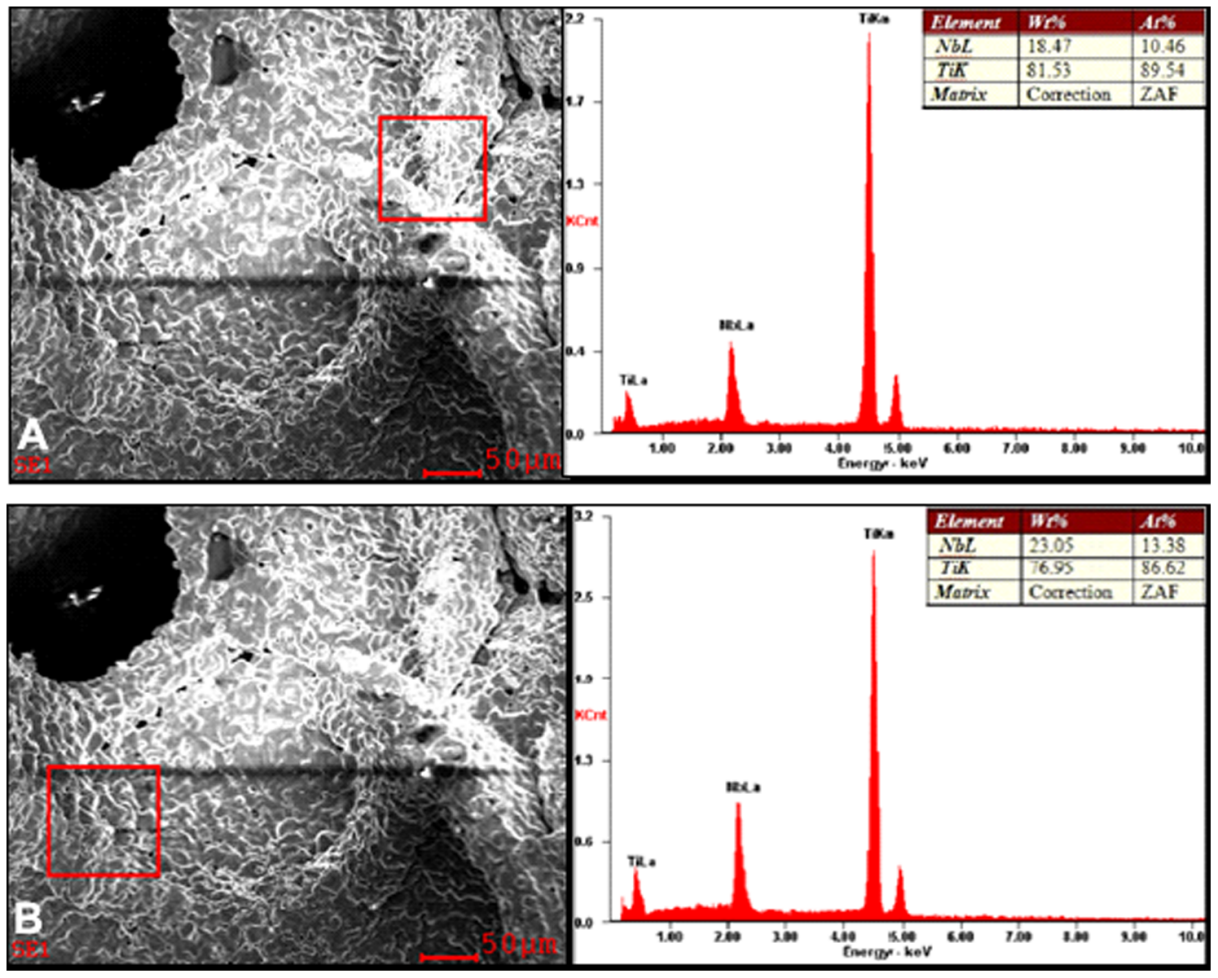
EDS analysis of alloy specimens. (A–B) EDS analysis of different regions of porous Ti-25Nb alloy.

Results of energy spectrum analysis show that the main elements on the surface of the porous titanium–niobium alloy were titanium and niobium, in which the strongest signal was at 4.5 keV with the second peak at 2.2 keV. The surface of the porous titanium–niobium alloy was mainly composed of the two elements. The weight and atomic percentage of titanium were 76.95% to 81.53% and 86.62% to 89.54% respectively, whereas those for niobium were 18.47% to 23.05% and 10.46% to 13.38%, respectively (Figs. 8A to 8B).

### Mechanical Performance of Ti-25Nb Alloy

The values of elastic modulus of the porous titanium–niobium alloys of the dense group, 40% porosity group, and 70% porosity group were 4.57, 3.74, and 2.23 GPa, respectively. The compressive strengths of the dense group, 40% porosity group, and 70% porosity group were 212.7, 146.7, and 94.8 MPa, respectively.

### Rabbit BMMSCs

**Figure 9:**
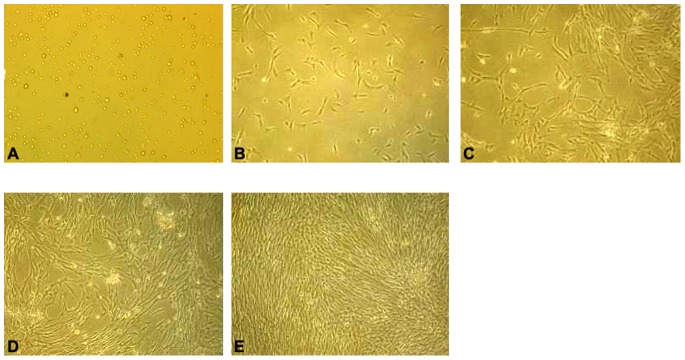
Inoculation, Culture and passage of Rabbit BMMSCs. (A) Primary BMSCs were inoculated, presented with round shape (magnification ×100). (B) There were a large number of adherent cells. Cell division could be seen at this phase (magnification ×100). (C) Within 4 to 5 days, the cells entered into logarithmic growth phase in which the amount increased and the cells arranged closely more like fibroid cells morphology (magnification ×100). (D) Within 8 to 10 days cell monolayer fused closely to 80% with swirl-like growth (magnification ×100). (E) The cells in the third generation were of the uniform morphology which grew with parallel or spiral arranging, and they became monolayer fused in 6 to 7 days (magnification ×100).

**Figure 10:**
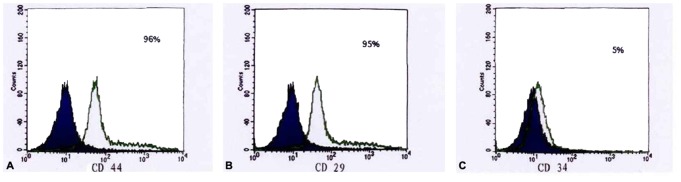
Identification of Rabbit BMMSCs. (A–C) The results of FACS respectively showed that 96% of the third-generation cells expressed CD44 and 95% of the cells expressed CD29, while only 5% of the cells expressed CD34.

After the primary rabbit BMMSCs were inoculated in the culture bottle, some round-shaped cells could be observed. Within 48 h, cell division and a large number of adherent cells were observed at this phase. Within 4 d to 5 d, the cells entered into the logarithmic growth phase, in which the amount of cells increased and the cells were arranged more closely, similar to fibroid cell morphology. Within 8 d to 10 d, the cell monolayer fused closely to 80% with a swirl-like growth. The cells were passaged by a proportion of 1∶3. A large number of passaged cells became adherent in 2 h to 3 h, and their growth rate became significantly faster. These cells exhibited a spindle-like fiber morphology. Third-generation cells showed a uniform morphology, which grew with a parallel or spiral arrangement. These cells fused into a monolayer in 6 d to 7 d (Figs. 9A to 9E). The surface antigen of rabbit BMMSCs were detected by FACS. The results show that 96% of the cells expressed CD44, 95% of the cells expressed CD29, and only 5% of the cells expressed CD34. The cell phenotype was uniform in populations of third-generation rabbit BMMSCs. This cell phenotype met the phenotypic characteristics of mesenchymal stem cells (Figs. 10A to 10C).

### Detection of Cell Adhesion and Proliferation

**Figure 11:**
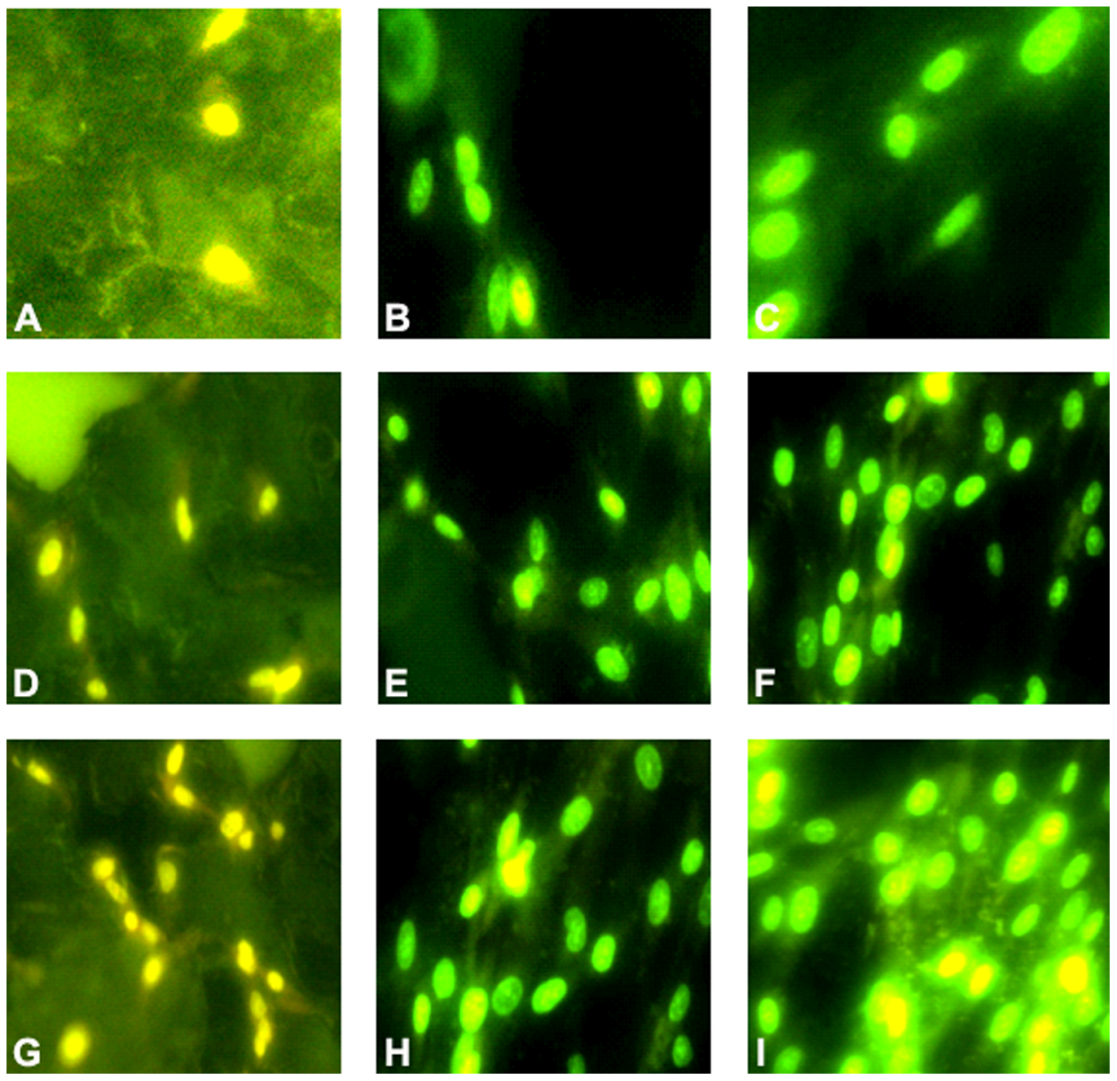
Detection of cell adhesion and proliferation after the cells were mixed with the alloy specimens. (A–I) The figures respectively showed cells proliferating in the compact group at the time point of 3 h, 24 h and 72 h. The cells all attached and proliferated well (magnification ×200). (D–F) They presented the condition of cells proliferating in the 40% porosity group at the time point of 3 h, 24 h and 72 h. The cells proliferating speed is faster than the compact group (magnification ×200). (G–I) The cells proliferating speed is fastest among the three groups, however, cells interconnected confluently in the 70% porosity group at the time point of 72 hours (magnification ×200).

Three hours after the cells were mixed with the alloy, fluorescent microscopy showed that the cells attached well in all groups. The cells presented fusiform, triangular, spherical, elliptical, and polygonal shapes. After 24 h, the round and polygonal cells grew well on the surface of materials, whereas the cells proliferated faster on the surface of materials in the 40% and 70% porosity groups. After 72 h, the cells proliferated well in all the groups, however, cells interconnected confluently in the 70% porosity group (Figs. 11A to 11I).

### Scanning Electron Microscopy

**Figure 12:**
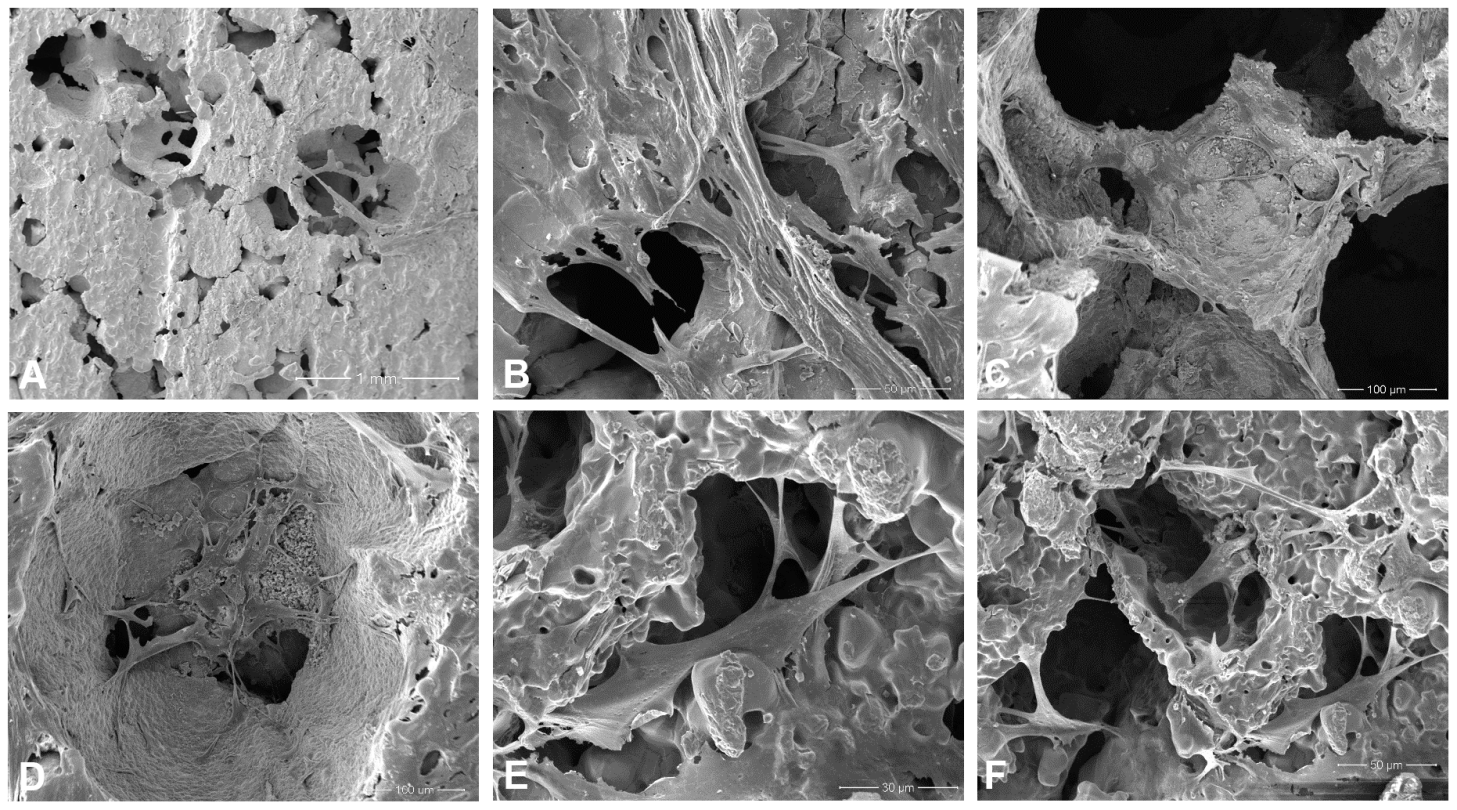
Scanning Electron microscopy of cells co-cultured with alloy specimens at different time point. (A) After the cells had been inoculated for 3 hours, rabbit bone marrow mesenchymal stem cells which were regular elongated-spindly shaped adhered to the surface of the alloy with 70% porosity. (B) After 24 hours, pseudopodia-like protrusions of the cells appeared, interrelated between each other with the cells tightly attached to the surface of the alloy with 70% porosity. (C–D) After 72 hours inoculated, cells began growing to the inside pores of the alloys with 70% porosity (C) and 40% porosity (D). (E–F) The cells were growing from the edge to inside of pores of the 70% porosity group in which extracellular matrix also could be seen.

After 3 h of inoculation, rabbit BMMSCs with regular, elongated, spindly shapes adhered to the surface of the materials. After 24 h, pseudopodia-like protrusions of the cells appeared, which were interrelated between each other with the cells tightly attached to the surface of the material. The cells on the surface of samples were highly concentrated in the 40% and 70% porosity groups. However, the cells were growing from the edge into the pores on the surface of the 70% porosity group, and extracellular matrix (ECM) could be observed (Figs. 12A to 12F).

### MTT Colorimetric Assay

The assay showed the absorbance (OD value) of rabbit BMMSCs, which were cultured in the leaching liquors of specimens with 50% and 100% concentration for 2, 4, and 7 d. The results were similar to the cells of the blank control group, which were cultured in culture medium (10% FBS+DMEM/F12+ BFGF). No significant differences between each time quantum (P>0.05) were observed. The absorbance values of each group increased on day 4 over the first 2 d, and on day 7 over the first 4 d (Table 7). The relative growth rate of cells on the materials was between 94% and 113%, with the toxicity level of materials from 0 to 1 (Table 7).

**Table 7:** Results of MTT tests for porous Ti-25Nb groups and control group.

Group	2 d culture			4 d culture			7 d culture		
	OD	RGR	Grade	OD	RGR	Grade	OD	RGR	Grade
Group A	0.198	100%	0	0.359	100%	0	0.926	100%	0
Group B	0.161	94%	1	0.380	106%	0	0.994	104%	0
Group C	0.188	95%	1	0.351	98%	1	1.046	113%	0

Group A: Blank control group; Group B: Leaching liquor with50% concentration group; Group C: Leaching liquor with 100% concentration.

### IL-6 Expression

**Figure 13:**
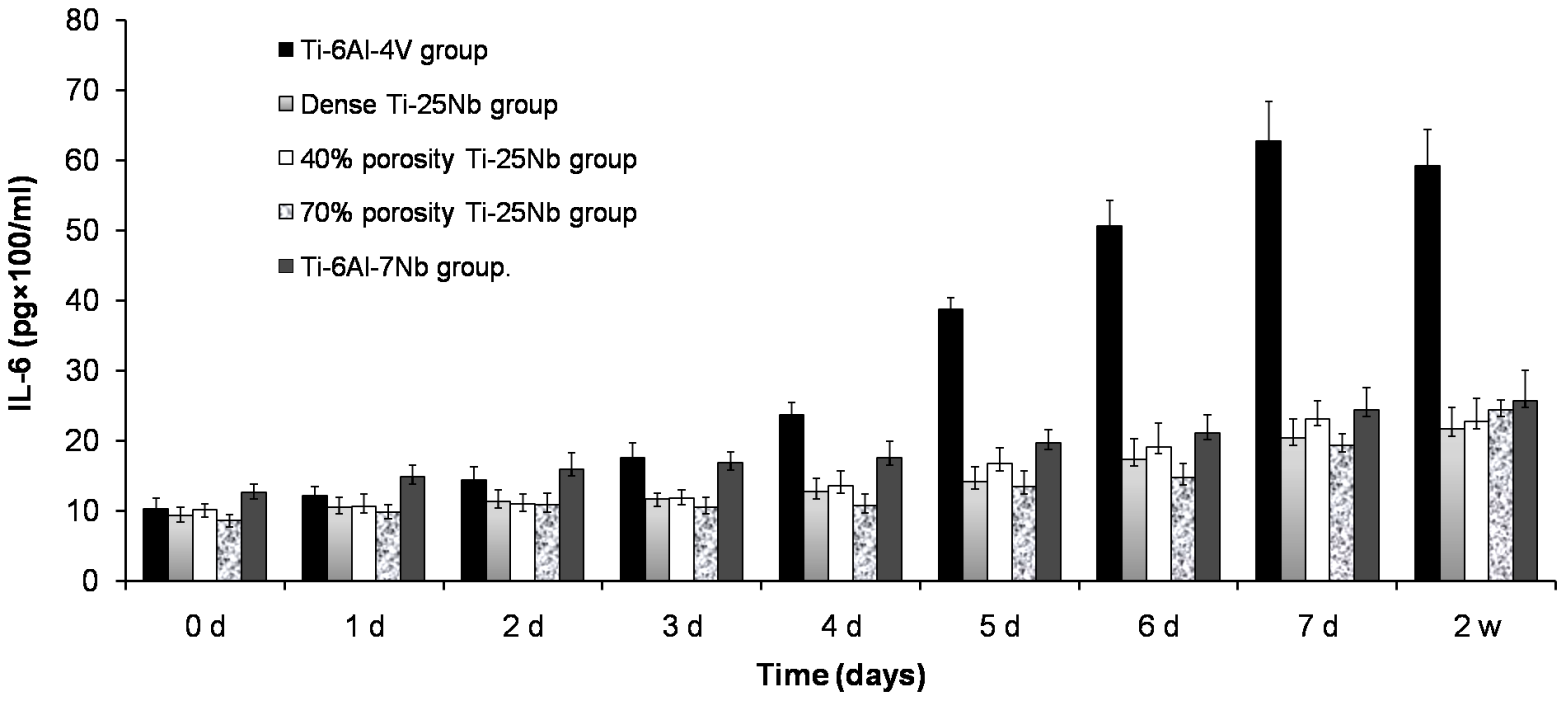
Comparison of IL-6 level in different groups.

No significant difference was found in each time point among the dense Ti-25Nb alloy, 40% porosity, 70% porosity groups and the Ti-6Al-7Nb group (P>0.05). However, the IL-6 content in the supernatant of Ti-6Al-4V alloy specimens increased gradually with the culture time. The difference was statistically significant compared with that of the other four groups (P<0.05, Table 8 and Fig. 13).

**Table 8:** Comparison of IL-6 levels in different groups at different time points (pg×100/ml).

Group	0 d	1 d	2 d	3 d	4 d	5 d	6 d	7 d	2 w
Group I	10.2±1.6	12.1±1.3	14.4±1.8	17.5±2.2	23.6±1.8	38.7±1.6	50.6±3.6	62.7±5.7	59.2±5.2
Group II	9.3±1.2	10.5±1.4	11.3±1.7	11.6±0.9	12.7±1.9	14.1±2.1	17.3±2.9	20.3±2.8	21.6±3.1
Group III	10.1±0.8	10.6±1.8	10.9±1.4	11.8±1.2	13.5±2.1	16.7±2.3	19.1±3.4	23.1±2.6	22.7±3.3
Group IV	8.6±0.8	9.8±1.0	10.8±1.7	10.5±1.4	10.7±1.7	13.4±2.2	14.7±2.0	19.3±1.7	20.5±1.4
Group V	12.6±1.2	14.8±1.7	15.9±2.3	16.8±1.6	17.5±2.4	19.7±1.8	21.1±2.6	24.4±3.1	25.7±4.3

Group I: Ti-6Al-4V group; Group II: Dense Ti-25Nb group; Group III: 40% porosity Ti-25Nb group; Group IV: 70% porosity Ti-25Nb group; Group V: Ti-6Al-7Nb group.

## Discussion

Irregular pore shapes, inappropriate pore size, inhomogeneous pore distribution and inhomogeneous elements distribution could occur during conventional powder metallurgy processing techniques. Almost all the fabrication routes suffer from the problems stated to a certain extent [20]. For clinical biological materials, especially alloys, the characteristics of their surfaces such as chemical composition, roughness, the internal pore size, and porosity are important. For our alloy, although inhomogeneous elements distribution existed, Ti-Nb alloy do not have affinity to the tissues, the internal pore size and porosity are the determining factors for cell adhesion and tissue ingrowth. For materials in orthopedic implants, mechanical properties influence the combination of biomaterials and their stabilities in vivo. The elastic modulus of the materials should satisfy the body weight of the necessary mechanical strength and the elastic modulus to bone. If these factors are not satisfied, stress shelter can cause implant loosening. The compressive strength and elastic modulus of biological materials need to be balanced to extend in-service life in vivo. Therefore, studies on the surface characteristics and mechanical properties of orthopedic implants are important.

Most of the currently available titanium and titanium alloy implants in clinical application are dense. Although some methods, such as surface treatment, can be applied to increase the contact area, these methods produce certain effects on bone integration between implants and bone. Wang [21] had already found that the osseointegration rate of the TiNb-coating alloy was better than the Ti-coating alloy, which indirectly proved the significance of the niobium mixed with titanium. However, the bone tissue can only extend to the implant surface field and not the interior of implants. Biological fixation is not achieved when long-term stability is not guaranteed. Given the difference in mechanical properties, the dense-type alloy causes stress shelter after implantation in the body, which eventually lead to bone resorption. Many studies showed that excess bone loss influences the long-term effect of implants and leads to implant displacement, aseptic loosening, fracture around the prosthesis, and increase in the difficulty of revision surgery [22]. The relationship between the bone loss and implant characteristics has been demonstrated from animal experiments and clinical follow-up, in which stress shelter is the key factor that induces the bone to the plastic process and bone morphology changes, which result in bone resorption [23]. Bobyn [24] experimented on bilateral total hip replacement with non-cemented type, and found that a relationship exists between the femoral prosthesis and bone resorption caused by hardness and stress of the implant. The study also showed that bone resorption in the porous design is significantly low than that in the compact design because of the better elasticity in the prosthesis of the porous design. Quantitative analysis of the results showed that the quality of femur by the side of the porous prosthesis is 25% to 35% more than that of the compact design. Sychterz [23] also found a close relationship between implant hardness and femoral bone resorption in a study of 20 cadavers with uncemented arthroplasty.

Solid-state titanium shows characteristics of heterogeneous transformation at a transition temperature of 882.5°C. Below this temperature, titanium is in the α-phase. Above this temperature, titanium transforms into β-phase. The transformation characteristics of titanium are closely related to purity. The addition of another metallic element into the titanium base can change the temperature of heterogeneous transformation in titanium, thereby affecting the phase composition of the titanium alloy at room temperature. Ti, Nb, Zr, Sn, Ta, and Fe are non-toxic or low cytotoxic elements that can improve the mechanical properties, corrosion resistance, and biocompatibility of the alloy. Non-toxic beta-stable elements such as niobium, tantalum, and zirconium alloys not only have good histocompatibility [25], but can also increase the strength and lower elastic modulus. Niobium can reduce the transition temperature for beta phase, increase the two-phase zone of alpha and beta, and improve the machinability and corrosion resistance of titanium alloy [26], [27]. Titanium alloys such as Ti-Nb-Ta–Zr,Ti-Nb-Zr-Sn, and Ti-Al-V alloys [28], [29] have high strengths and low elastic moduli, which are closer to those of the human cortical bone. These alloys have been widely used as artificial hip joints, bone implants, and dental implant materials in recent years. In our study, the Ti-25Nb alloy had high strengths and a low elastic modulus that was close to that of human bone.

Many researchers have focused on the relationships among porosity, pore diameter, and bone growth behavior. However, a consensus for the optimal pore size and porosity has not been reached. In general, porous materials with apertures from 150 µm to 600 µm can allow bone ingrowth [30], [31], [32], [33]. The SEM results show that the alloy in our study was porous, with a diameter of about 200 µm to 500 µm and three-dimensional connectivity between pores. However, the elastic modulus and mechanical strength of porous metal materials increased with the reduced porosity. Therefore, a balance between the two could result in better implant performance. Porous metals, which have proper mechanical strength and high porosity, require the dense materials around them to have high mechanical strength. In our study, the 70% porosity titanium alloy had a high compressive strength of 94.8 MPa close to cortical bone and an elastic modulus of 2.23 GPa, which was closer to cancellous bone. Hence, the effect of stress shelter could be alleviated to a large extent by our alloy.

Rabbit BMMSCs were selected for comprehensive cytotoxicity evaluation of all titanium–niobium alloys in our experiments, which simulated the implantation of biomaterials into the marrow cavity in vivo. Rabbit BMMSCs were cultured with different concentrations of leaching solution of porous titanium–niobium alloy material to reflect the reactions of possible toxic substances to the cell. The experiment could detect the effects on the number of active cells and metabolic functions, and was highly sensitive to the toxicity of the alloy material based on the extent of cell damage. Our experimental results show that the leaching solution of each group with different concentrations had no poisonous reaction to the cells, which presented good proliferation on the surface of specimens and accounted for good biocompatibility of the alloy.

To satisfy the clinical requirements, biological materials must provide adequate internal space and surface area for cell growth to facilitate the adhesion and proliferation of cells, as well as the formation and deposition of the ECM. Porosity and pore diameter are two of the most important parameters in biological porous materials that directly affect the interaction between cells. The materials with pore diameter of at least 100 µm can provide cells with a large area for cell adhesion. These cells can also promote nutrient, gas, and waste exchange between the cells and the surrounding environment. Porosity can affect the area of cell adhesion, whereas the pore size and connectivity between pores influence the access and distribution of cells, nutrient supply, and production of metabolic products. We obtained cellular-connected specimens of 70% porosity with a diameter of 550 µm to 650 µm, which was suitable for cells. Nonyl–acridine orange staining and SEM were used to observe the adhesion and proliferation of cells on the surface of materials in the dense-type and different porosity groups. Results show that cell proliferation on the surface of materials in the porous group was significantly higher than the smooth-faced dense group at different time points. The cells grew best on the surface of samples in the 70% porosity group, in which some cells even grew deeply into pores. Therefore, we infer that this phenomenon was mainly due to the roughness of the material. Materials that are sprayed and treated with acid have rough surfaces, which favor biological cell responses [34], [35]. The surface of the porous titanium–niobium alloy (Ra: 30 µm to 40 µm) used in our experiment was rougher than that with spraying and acid treatment on the surface (Ra: 3 µm to 4 µm) [36], which possibly improved cell reactions. In addition, the rough surface of the material can increase the bonding strength between bones and implant [37]. Osteoblasts can adhere to rough surfaces faster [38]. Roughness of a hydroxyapatite ceramic surface can improve the adhesion and proliferation of cells with higher surface roughness, resulting in larger cell adhesion strength [39], [40]. The chemical composition, morphological structure, roughness, corrosion resistance, surface energy, and crystal structure of biological materials have been confirmed to affect cell behaviors [41], [42]. The surface properties of materials can influence cell reactions mainly by changing the cytoskeleton and extending the filament network structure of cytoplasmic protein in eukaryotic cells [43]. Porous three-dimensional scaffolds serve as the temporary ECM for cell adhesion, growth, and differentiation [44], [45]. The interaction between the ECM proteins and cells can directly control cell behaviors, such as adhesion, migration, proliferation, differentiation, and apoptosis [46]. Porous titanium scaffold has good biocompatibility in the treatment of bone defects. The scaffold can support the internal growth of bone tissue through the interconnected voids [47]. In our study, the proliferation of rabbit BMMSCs on the surface of Ti-25Nb alloys with 40% and 70% porosity was better than that in the compact group. Therefore, the porous design was more advantageous for bone tissue growth. Porous designs are conducive for cell ingrowth, nutrient supply, and waste discharge of cells.

Our study also tested the IL-6 concentrations of the culture supernatants, which were cells with Ti-6Al-4V alloy, Ti-6Al-7Nb alloy, compact–type Ti-25Nb alloy, Ti-25Nb alloy with 40% porosity, and Ti-25Nb alloy with 70% porosity, at different time points. IL-6 is a sensitive factor in the inflammatory response [48]. IL-6 reacts in almost all cells in the body (including fat cells and osteoblasts). High levels of serum IL-6 can indicate early transplantation-related complications [48]. IL-6 was mainly activated in the acute reaction phase and generally increased with increasing temperature. High body temperature is the automatic defense response of the body against pathogens. However, when the cells and biomaterials are combined, the elevated temperature will affect their interaction, which is unfavorable for early bone ingrowth in biological materials, ultimately affecting the stability of the implanted biomaterials. A researcher used biopsy of 29 cases with bone cement hip replacement, and compared it with inflammation (IL-6 levels) of methyl methyl acrylic acid salt in vitro, and found that both are positively correlated [49]. In the present study, the IL-6 concentration of each cell culture supernatant in different groups was determined. The results show that the release of IL-6 in the Ti-25Nb alloy group was lower than that in the Ti-6Al-4V alloy, which indicates that the Ti-25Nb alloy did not increase immunogenicity. No obvious difference in the IL-6 concentration was observed among the three groups of Ti-25Nb alloys and Ti-6Al-7Nb alloy, which illustrates that the porous design did not cause excessive inflammatory reaction. Though no significant difference in the IL-6 concentration between Ti-Nb alloy and Ti-Nb-Al alloy, the element Al had been associated with neurological disorders [50].

## Conclusion

All porous titanium–niobium alloys showed good biocompatibility regardless of the percentage of porosity. The basic requirement of clinical orthopedic implants was satisfied, which makes the alloy a good prospect for biomedical application. The alloy with 70% porosity had the optimum mechanical properties, as well as suitable pore size and porosity, which allowed more bone ingrowth.

## Supporting Information

Figure S1 **A porous Ti-25Nb alloy specimen with 70% porosity designed according to Rabbit femoral medullary cavity.**
(JPG)

Figure S2 **A porous Ti-25Nb alloy specimen with 40 porosity designed according to Rabbit femoral medullary cavity.**
(JPG)

Figure S3 **A porous Ti-25Nb specimen with 70% porosity was implanted into proximal femoral medullary cavity of a rabbit.**
(JPG)

Figure S4 **After the rabbit femoral cortical bone on one side was open longitudinally, close integration could be seen between porous Ti-25Nb implant and bone, solid, osteoporosis was difficult to remove from on the surface of the porous implant.**
(JPG)

Figure S5 **Calcium salt deposits could be seen from the surrounding soft tissue (HE×200).**
(JPG)

Figure S6 **X ray of 40% porosity group after two weeks of the implantation.**
(JPG)

Figure S7 **Calcium deposits can be seen on the surface of Ti-25Nb alloy specimens with 70% porosity by SEM.**
(PNG)

Figure S8 **Pulling out tests of specimens in bone.**
(JPG)
