# Supplementary figures and images for: Potential Use of Porous Titanium–Niobium Alloy in Orthopedic Implants: Preparation and Experimental Study of Its Biocompatibility In Vitro
Source: PLoS One. 2013 Nov 19;8(11):e79289. doi: 10.1371/journal.pone.0079289 (PMC3834032; doi:10.1371/journal.pone.0079289)

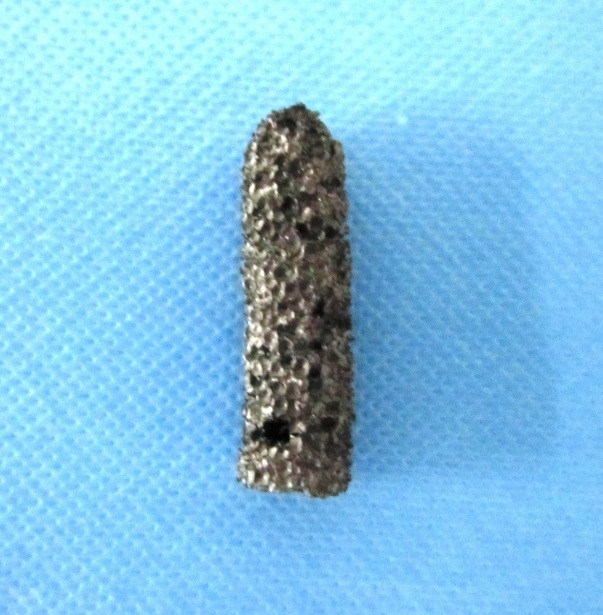

Supplement: Figure S1 — A porous Ti-25Nb alloy specimen with 70% porosity designed according to Rabbit femoral medullary cavity. (JPG) [file pone.0079289.s001.jpg]

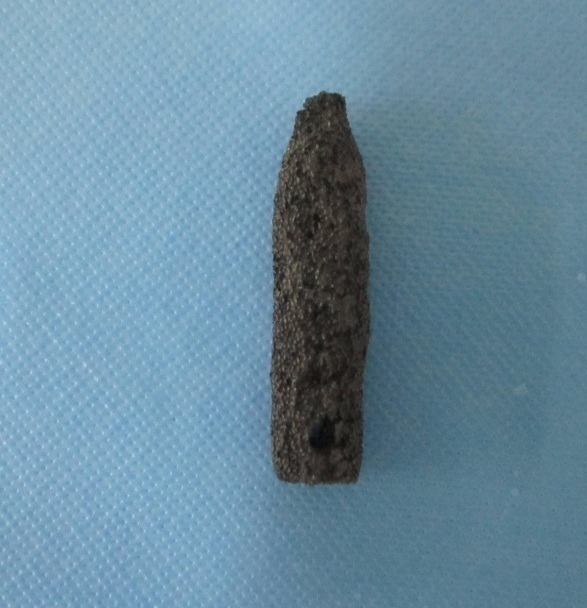

Supplement: Figure S2 — A porous Ti-25Nb alloy specimen with 40 porosity designed according to Rabbit femoral medullary cavity. (JPG) [file pone.0079289.s002.jpg]

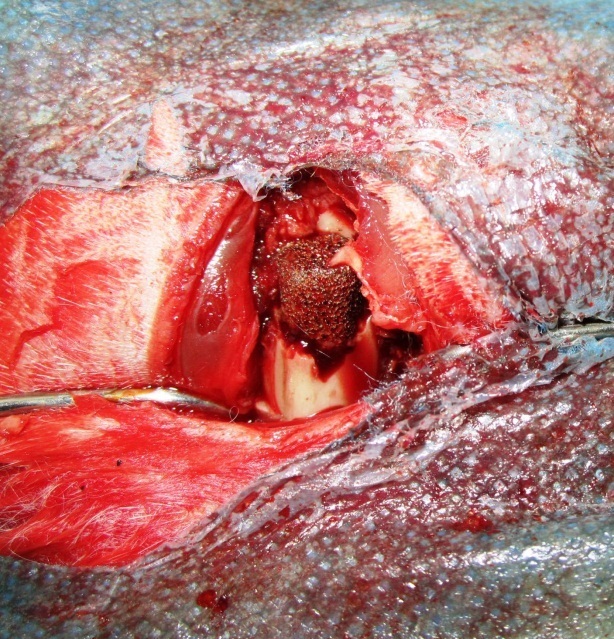

Supplement: Figure S3 — A porous Ti-25Nb specimen with 70% porosity was implanted into proximal femoral medullary cavity of a rabbit. (JPG) [file pone.0079289.s003.jpg]

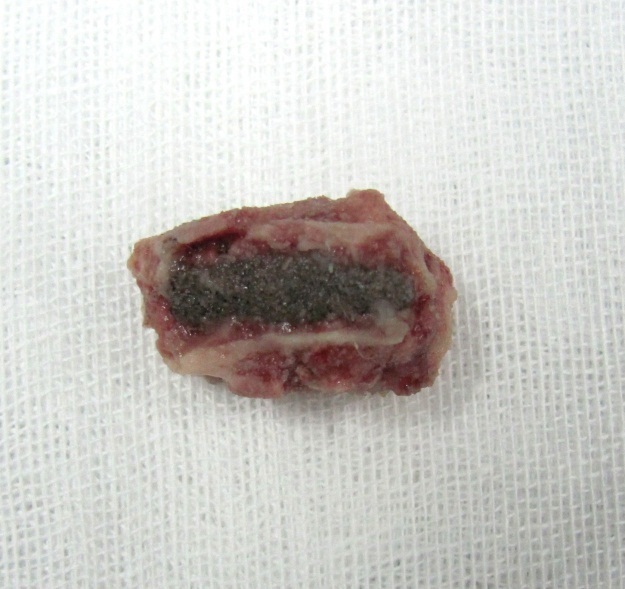

Supplement: Figure S4 — After the rabbit femoral cortical bone on one side was open longitudinally, close integration could be seen between porous Ti-25Nb implant and bone, solid, osteoporosis was difficult to remove from on the surface of the porous implant. (JPG) [file pone.0079289.s004.jpg]

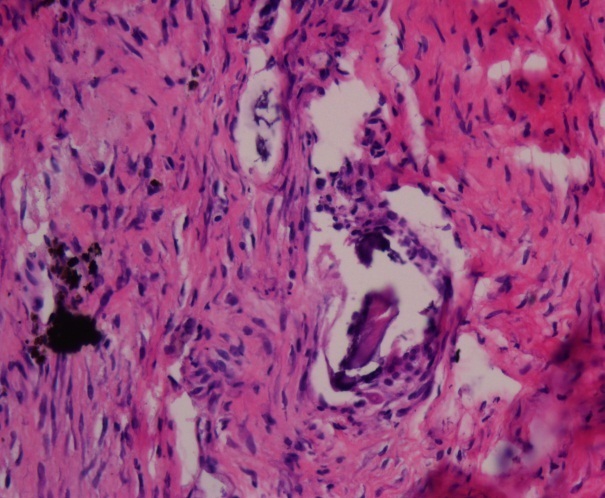

Supplement: Figure S5 — Calcium salt deposits could be seen from the surrounding soft tissue (HE×200). (JPG) [file pone.0079289.s005.jpg]

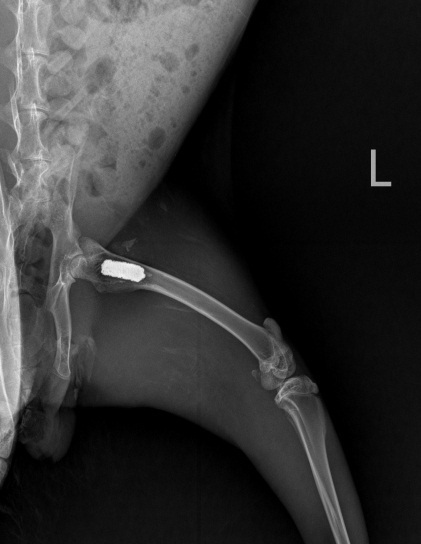

Supplement: Figure S6 — X ray of 40% porosity group after two weeks of the implantation. (JPG) [file pone.0079289.s006.jpg]

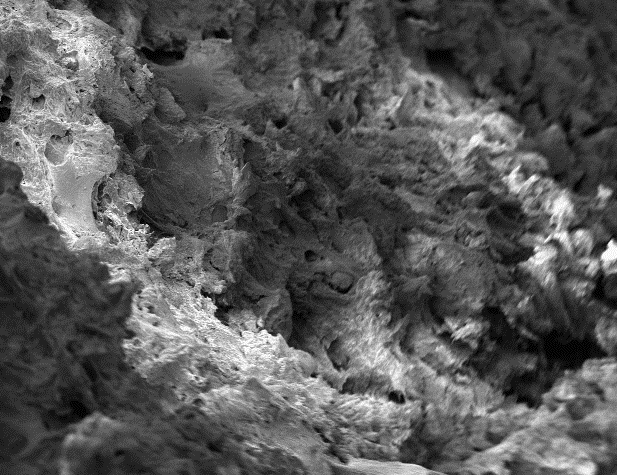

Supplement: Figure S7 — Calcium deposits can be seen on the surface of Ti-25Nb alloy specimens with 70% porosity by SEM. (PNG) [file pone.0079289.s007.png]

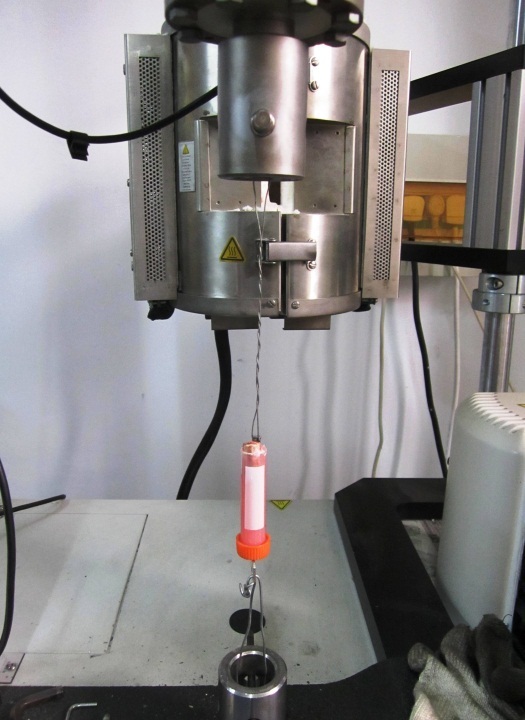

Supplement: Figure S8 — Pulling out tests of specimens in bone. (JPG) [file pone.0079289.s008.jpg]
